# Supplementary material for: Cloud BioLinux: pre-configured and on-demand bioinformatics computing for the genomics community
Source: BMC Bioinformatics. 2012 Mar 19;13:42. doi: 10.1186/1471-2105-13-42 (PMC3372431; doi:10.1186/1471-2105-13-42)
Supplement: Additional file 1 — Supplementary 1 Cloud BioLinux software documentation in the form of a mini, self-contained website. Users need to download and uncompress the .zip file, and open through a web browser the "index.html" file available on the main directory. (ZIP 1823 kb). [file 1471-2105-13-42-S1.ZIP › Cloud-BioLinux-Package-Documentation/docs/bioruby.html]

Bio-Linux Software Documentation Pages

Back to search form

## bioruby

|  |  |
| --- | --- |
| Name | bioruby |
| Description | **BioRuby** is a project that aims to implement an integrated environment for Bioinformatics with Ruby language. Design philosophy of the BioRuby library is KISS (keep it simple, stupid), to maximize the usability and the efficiency for biologists as a daily tool.  The project was started in Japan and supported by University of Tokyo (Human Genome Center), Kyoto University (Bioinformatics Center) and the Open Bio Foundation. |
| Homepage | http://bioruby.org/ |
| Remote Documentation | http://wiki.bioruby.org/English/ |

|  |
| --- |
| Please see Remote Documentation |
